# Graves’ ophthalmopathy: the clinical and psychosocial outcomes of different medical interventions – a systematic review

**DOI:** 10.1136/bmjophth-2023-001515

**Published:** 2024-06-17

**Authors:** Oyinlola Maria Bello, Maralyn Druce, Ejaz Ansari

**Affiliations:** 1 Maidstone and Tunbridge Wells NHS Trust, Maidstone, UK; 2 Queen Mary University of London William Harvey Research Institute, London, UK; 3 Canterbury Christ Church University Institute of Medical Sciences, Chatham, UK; 4 Ophthalmology, Maidstone and Tunbridge Wells NHS Trust, Maidstone, UK

**Keywords:** Ocular surface, Orbit, Inflammation, Immunology, Muscles

## Abstract

**Background:**

Graves’ ophthalmopathy is a complex autoimmune disorder that can significantly affect quality of life (QoL), vision and physical appearance. Recently, a deeper understanding of the underlying pathogenesis has led to the development of novel treatment options.

**Aims:**

The purpose of this review is to explore the current literature on conventional and novel treatment modalities and to evaluate which interventions provide the most favourable psychological and clinical outcomes in patients with moderate to severe, active Grave’s ophthalmopathy. For example, QoL is an important psychosocial outcome of disease management. However, available literature demonstrates that not all clinically effective treatment options improve patients’ QoL.

**Methods:**

A systematic literature review was conducted to assess the clinical and psychosocial outcomes of different therapies for Graves’ ophthalmopathy. An extensive database search of Ovid Medline, Ovid Embase and Cochrane Central Register of Controlled Trials was conducted. Studies generated were reviewed and the relevant selected data were retrieved and analysed.

**Results:**

Results showed intravenous steroids, rituximab (RTX), tocilizumab and teprotumumab were all significantly effective in improving Clinical Activity Scores. Orbital radiotherapy showed a slight improvement in proptosis and diplopia. All interventions were safe with few serious adverse events being reported across all studies. All treatment modalities demonstrated beneficial improvements in both components of the Graves’ Ophthalmopathy-QoL (QoL) questionnaire, apart from orbital radiotherapy which only demonstrated improvements in the visual functioning subscale. Teprotumumab was identified to be the most effective intervention for improving both clinical and psychosocial outcomes. However, further research needs to be conducted to evaluate its side effect profile and cost-effectiveness. Nonetheless, with time it has the potential to be a first-line treatment option in the management of active moderate to severe Graves’ ophthalmopathy.

**WHAT IS ALREADY KNOWN ON THIS TOPIC**

- According to the European Group of Graves Orbitopathy (GO) guidelines, intravenous methylprednisolone in combination with mycophenolate sodium is the recommended first-line treatment option for the management of moderate to severe GO.

**WHAT THIS STUDY ADDS**

- This study reviewed conventional therapies and novel biological treatments to evaluate which treatment modalities are the most clinically effective and have the best psychosocial outcomes.

**HOW THIS STUDY MIGHT AFFECT RESEARCH, PRACTICE OR POLICY**

- Teprotumumab was identified to be the most effective intervention, although it is still not currently licensed in the UK for the management of GO, therefore, additional research on the drug is encouraged.

## Introduction

Graves’ orbitopathy (GO) is a sight-threatening disease characterised by visual functional deficit and social impairment. Proptosis, strabismus, diplopia and the well-known ‘thyroid eye’ appearance are caused by soft tissue expansion, adipocyte proliferation, extraocular muscle enlargement and eyelid retraction. These clinical manifestations can cause increased tear evaporation due to extraocular exposure, which can trigger corneal epithelial damage and keratopathy. All of which may lead to reduced visual acuity and physical disfigurement in patients. Furthermore, due to the ophthalmic manifestations of GO, sufferers typically report an impact on their quality of life (QoL), as they are not able to carry out their regular daily activities such as driving, reading, watching television or vocational work. In addition to that, changes in appearance can cause instances of discrimination and psychological distress in social situations, making it difficult to maintain social relationships. Unfortunately, while medical and surgical intervention improves the progression of the disease, it may still impart a permanent physical disfigurement and functional disability that has a detrimental influence on patients’ psychosocial welfare and QoL.^1^

According to the WHO ‘Health is a state of complete physical, mental and social well-being, not merely the absence of disease or infirmity’.^2^ QoL assessments associated with health states have become increasingly important in recent years and QoL instruments are included as a therapeutic outcome in most clinical trials today. A physician’s ultimate concern when treating a patient is the well-being of the whole person rather than the sole improvement in clinical parameters. For example, small improvements in proptosis, soft tissue swelling or eyelid retraction may have no impact on a patient’s well-being if they are still burdened with severe diplopia or the adverse effects of treatment.

This poses an issue because some of the conventional recommended treatment options in the management of active moderate to severe GO are not disease-specific and are known to have little or no impact on a patient’s QoL after treatment.^3^ Sir William Osler famously said, ‘The good physician treats the disease; the great physician understands the patient and the context of the patient’s illness’.^4^

Patients with Graves’ eye disease have been known to report concerns about their appearance and difficulties in dealing with social situations following their diagnosis, and there is evidence to suggest that these concerns and difficulties continue long term.^5^ Therefore, it is in the best interest of the patient that the treatment options prescribed are not only clinically effective but also beneficial in improving well-being. There are several studies available that have evaluated the changes in quality-of-life scores after management with conventional treatment options such as oral or intravenous steroids and orbital radiotherapy.^6–8^ Wickwar *et al* conducted a systematic review evaluating the psychosocial outcomes of different medical and surgical treatment options in the management of thyroid eye disease. The researchers concluded that their study was limited by the quality of papers included but identified that management with intravenous steroids and orbital decompression surgery had long-term favourable effects on psychosocial outcomes.^9^ Since then, further understanding of the pathogenesis of GO has led to the development of a new wave of targeted therapies using biologics. Recent clinical trials have shown that treatment with new biologics such as teprotumumab and tocilizumab are clinically effective and improve QoL scores.^10 11^

## Aims and objectives

The primary aim of this systematic review was to measure the improvement in quality-of-life scores before and after treatment with biologics (tocilizumab, teprotumumab and rituximab) compared with conventional treatment options (glucocorticoids and orbital radiotherapy) in the management of active moderate to severe Graves’ ophthalmopathy, to establish which treatment options, have the most beneficial effects on patients’ well-being. The secondary aims of the study were to compare the overall clinical effectiveness of the different treatment modalities; specifically looking at outcomes such as adverse effects, improvements in proptosis, diplopia and Clinical Activity Score (CAS).

### Methods and materials

### Research question

To carry out a comprehensive literature search the ‘PICO’ (Population, Intervention, Comparison, Outcome) tool framework was used to formulate the clinical question that needed to be answered. The PICO question was as follows:

‘Do biologics provide more beneficial psychosocial and clinical outcomes compared with steroids and orbital radiotherapy in the management of active moderate to severe Graves’ ophthalmopathy?’

### Inclusion and exclusion criteria

#### Participants

Only studies reviewing adults over the age of 18 with active moderate-severe Graves’ Ophthalmopathy were included in this review. All other studies investigating patients outside of the specific scope were excluded.

#### Interventions

Monoclonal antibodies that could be prescribed according to the European Group on Graves Orbitopathy (EUGOGO) 2021 guidelines as monotherapy for the management of active moderate to severe Graves’ eye disease. This included rituximab (humanised monoclonal antibody against CD20), tocilizumab (humanised monoclonal antibody against IL-6) and teprotumumab (humanised monoclonal antibody against IGF-1R).

#### Comparators

First-line and second-line conventional monotherapy treatment options that are recommended by the EUGOGO 2021 guidelines: glucocorticoids and orbital radiotherapy, respectively. Standard of care is defined by trials or with a placebo. Interventions that could be used as an adjuvant such as ciclosporin or azathioprine, to improve outcomes were excluded. Local symptom treatment options such as eye-drops, gels and artificial tears were excluded.

#### Outcome measures

The primary outcome measure included improvement in the quality-of-life score. Secondary outcome measures included incidence of adverse events, improvement in the CAS, improvements in proptosis and improvements in diplopia.

#### Study types

Randomised control trials and prospective clinical trials or cohort studies of any design with no restrictions on language were included in this trial. Secondary forms of research such as literature reviews, systematic reviews or meta-analyses were excluded. Retrospective studies and case studies were also excluded.

#### Search strategy and data extraction

A detailed search was conducted of publications in the literature on this topic from January 2000 to 6 June 2022 (date of the search). All studies were selected from an electronic database search of Ovid Medline, Ovid Embase and CENTRAL (Cochrane Central Register of Controlled Trials) . All the studies gathered through the electronic database search were downloaded to the EndNote V.20 reference manager and then uploaded to the Covidence systematic review software, where the studies were screened against the inclusion and exclusion criteria.

Preferred Reporting Items for Systematic Reviews and Meta-Analyses statement can be found in online supplemental figure 1. 10 studies were retrieved and selected, based on the criteria outlined (online supplemental table 1). The Downs and Black quality assessment tool was used to critically appraise all the included studies^12^ (detailed in Supplementary data file). The mean quality assessment score was 21.7/28. Data extraction was guided by the review question and a data extraction template was generated to extract relevant information from the selected studies (online supplemental tables 2 and 3).

10.1136/bmjophth-2023-001515.supp1 Supplementary data

10.1136/bmjophth-2023-001515.supp2 Supplementary data

## Results

Management with intravenous methylprednisolone (IVMP) and teprotumumab both significantly improved patients’ CASs (p<0.001), treatment with IVMP did not significantly improve patients’ diplopia in any of the studies selected and only a minority of patients showed improvements in proptosis. Orbital radiotherapy demonstrated slight non-significant improvements in proptosis and diplopia at p=0.26 and p=0.36, respectively. Teprotumumab was the only treatment option to show significant improvement in both diplopia and proptosis (p<0.001). However, 81.5% of patients treated with teprotumumab experienced adverse effects compared with 27% of patients who were managed with IVMP.

Orbital radiotherapy was found to not have a significant impact on the overall patients’ QoL scores. There was an improvement in the visual functioning subscale (p=0.05), however, there was minimal improvement in the appearance subscale (p=0.61). Although only one study was generated from the search other published studies also confirm similar findings.^3 8^ Intravenous steroids were found to be effective in gaining rapid control and improving clinical activity and QoL scores. Treatment with the highest dose (7.47 g) demonstrated the most favourable outcomes, however, it was associated with the highest incidence of serious adverse effects. Hence, why 4.5 g is the recommended dose.^13^

In the IVMP versus RTX study,^14^ both treatment arms showed similar long-term beneficial outcomes in the clinical activity (p<0.006) and QoL scores. However, there was no significant improvement in diplopia or proptosis in both therapeutic groups. A greater percentage of patients treated with rituximab (86.6%) had adverse effects compared with the IVMP group (62.5%). These findings demonstrate that rituximab is not superior to IVMP and should only be used as a second line in patients who are unable to be managed with intravenous steroids.

Tocilizumab was investigated in patients with active GO who were steroid resistant.^11^ The findings showed that treatment with TCZ significantly improved CAS and proptosis at 16 weeks, however, at 40 weeks, there was no significant difference between the tocilizumab group and the placebo group indicating that the clinical improvements were not sustained. Benefits in quality-of-life scores were also noted, however, no improvement in diplopia was seen in either the treatment arm. Unfortunately, the results of the study cannot be extrapolated to evaluate if tocilizumab is more beneficial compared with steroids or orbital radiotherapy as the study was only conducted on patients with steroid-resistant GO.

## Discussion

This review has also shown that there is a relationship between disease activity and psychosocial outcomes, with improvement in CAS being positively correlated with GO-QoL scores in all studies. The relationship between quality-of-life scores and disease severity was difficult to obtain as the complete ophthalmological examination including eyelid measurements, corneal involvement, soft tissue involvement and visual function were not outcomes measured in this study. In addition to that, besides teprotumumab, none of the other treatment options significantly improved both strabismus and proptosis, factors which contribute to disease severity.

This systematic review aimed to determine if biologics provided more beneficial psychosocial outcomes compared with steroids and orbital radiotherapy in the management of active moderate to severe Graves’ ophthalmopathy. It also reviewed the clinical outcomes of different treatment modalities and assessed if improvements were sustained over a prolonged period.

This systematic review was limited due to a few factors; first, there was a range of different study designs with varied quality index scores. The reporting of patients’ selection and whether patients were representative of the population varied between studies. Most studies also failed to report the statistical power of the research. Unsurprisingly, the randomised control trials had higher-quality index scores compared with the prospective trials. However, due to the strict inclusion and exclusion criteria and limited research available, not a lot of studies were generated when ‘only RCTs’ were a part of the inclusion criteria, therefore, prospective studies were also included to broaden the search. A strength, however, noted across most studies was that regardless of the study design, researchers recognised the effects of confounding factors such as smoking and made adequate adjustments for these factors in the statistical analysis when calculating the results. Also, published studies and reviews within the field have shown similar findings to this systematic review.^15 16^

Another limitation was that QoL was not a primary outcome for most studies, therefore, when reporting outcomes some researchers focused more on the primary objectives and did not adequately display results from QoL scores.^17 18^ In the future potentially only retrieving studies that have QoL as the primary outcome may combat this limitation. There are, however, limited studies available with QoL as a primary outcome, therefore, broadening the inclusion criteria study designs will be necessary to be able to evaluate more research. An additional drawback was that this study only reviewed patients with moderate to severe active GO, therefore, further research into other patient cohorts could be beneficial and provide additional information. For example, treating patients with mild Graves’ ophthalmopathy using disease-modifying biologics could be more beneficial, effective and prevent the disease from worsening to the sight-threatening form of GO.

Furthermore, this study uncovered some limitations in a few of the assessment instruments used in the evaluation of GO. For example, the challenges associated with using generic Health-related quality of life (HRQL) questionnaires to assess therapeutic outcomes or, by equating CASs with disease severity. By highlighting this, it encourages physicians to be more cognizant of the shortcomings of different assessment tools and modify their practice accordingly to enhance patient evaluation and outcomes.

Lastly, many researchers failed to address or consider the degree of improvement in clinical activity or QoL scores that could have been attributed to disease burnout rather than treatment intervention. The average follow-up across studies was completed at 6 months, however, the duration of the active phase of GO, lasts on average from 3 to 18 months,^19^ meaning some patients could have stabilised and entered the quiescent phase of the disease by the time of the follow-up assessment thus influencing the results.

On another note, there were many strengths of this review. First, a systematic literature review was the method used to answer the research question. This involved a comprehensive search of multiple databases and a criterion-based selection of the relevant studies, as well as a rigorous critical appraisal and analysis of each selected study before overall conclusions were drawn. Using this approach has many advantages over other research methodologies in that it allows for transparency, accuracy, replicability and a reduced risk of bias.

Another advantage of this study was that it emphasised the importance of evaluating the psychosocial well-being of patients with GO. It also highlighted the importance of incorporating QoL assessments before and after therapeutic interventions and educating patients on how their QoL may change with different therapies.

Finally, this systematic review evaluates both the psychosocial and clinical outcomes of novel biologics compared with conventional therapies in the management of moderate to severe active GO. Overall, it was concluded that teprotumumab was the most effective intervention for improving disease activity, severity and QoL scores. These findings advance the field of GO since they can be used as a starting point for additional research. For instance, evaluating teprotumumab’s therapeutic efficacy in different cohorts of GO patients or comparing its efficacy when used alone versus in combination with other biologics or conventional treatments.

In summary, findings from this systematic review demonstrate that while biologics are effective in the management of GO. Teprotumumab is the only biologic that provides more beneficial psychosocial and clinical outcomes compared with steroids and orbital radiotherapy. This is mostly due to the IGF-1R inhibitor being able to significantly improve both diplopia and exophthalmos alongside clinical activity and QoL scores.^10 20^

## Conclusion

This systematic review evaluated the impact of different novel biologics versus standard conventional treatment options on quality-of-life outcomes and clinical effectiveness. This present review has identified that most treatment options apart from orbital radiotherapy have a beneficial impact on quality-of-life scores, with teprotumumab providing the most favourable, psychosocial and clinical outcomes combined.

While teprotumumab provided more beneficial psychosocial and clinical outcomes compared with steroids and orbital radiotherapy. It would be interesting to see its effectiveness in different patient cohorts, for example, in patients with mild or chronic Graves’ eye disease. Further research needs to be conducted on the long-term clinical effects of teprotumumab, evaluating its effect on reducing the need for strabismus and cosmetic surgery. Additional data collection and statistical analysis should also be conducted to determine teprotumumab’s impact on a patient’s quality-adjusted life years (QALY). QALY is the leading metric used to perform cost-effectiveness analysis, however, it is not without its controversies. First, the concept of assigning a value to perfect health is morally questionable. In the UK, the National Institute for Health and Care Excellence assigns a value of £20 000–£30 000 per QALY, which is generally defined as a year of perfect health.^21^ However, a perfect state of health does not equate to a more valuable life. For example, a person who is wheelchair-bound can live happily or even more happy compared with someone who is not. Also, contextual factors such as mental health are generally not considered when calculating QALYs. This is a limiting factor as a large aspect of Graves’ eye disease is associated with the negative psychosocial burden that patients experience. Therefore, additional research should analyse other metrics such as overall ophthalmic outcomes and disease-specific GO-Qol scores in determining cost-effectiveness. Nonetheless, because teprotumumab does significantly improve patients’ QoL scores and could potentially reduce the need for surgery (and the cost associated with surgery) it is likely to increase the overall QALY value. So, calculating cost-effectiveness using this metric could still be beneficial. However, teprotumumab’s side effect profile, clinical effectiveness and cost-effectiveness would still need to be compared with other available therapies before it is recommended as a first-line treatment option in the management of active moderate to severe Graves’ ophthalmopathy.

Currently, GO-QoL questionnaire is the most frequently used QoL questionnaire in clinical trials. Nevertheless, it is not without its limitations, for example, it does not evaluate a patient’s overall QoL score. The TED-QOL however, evaluates the overall QoL as well as visual functioning and appearance. Therefore, it would be recommended for research to be carried out evaluating both questionnaires to identify which disease-specific questionnaire is best correlated with disease severity.

With the growing recognition that quality-of-life outcomes are an essential component of therapeutic efficacy, it is recommended that more trials include disease-specific QoL questionnaires as a primary outcome measure. It is equally as important and recommended for physicians to carry out routine QoL assessments on their patients during consultations. This should be done, to be able to identify patients that may benefit from extra psychological support and to be able to consider psychological wellness scores when planning management. It is also important for patients to be educated on different management options and to be informed that not all clinically effective treatments will improve their QoL.
