# Supplementary figures and images for: Graves’ ophthalmopathy: the clinical and psychosocial outcomes of different medical interventions – a systematic review
Source: BMJ Open Ophthalmol. 2024 Jun 17;9(1):e001515. doi: 10.1136/bmjophth-2023-001515 (PMC11184183; doi:10.1136/bmjophth-2023-001515)

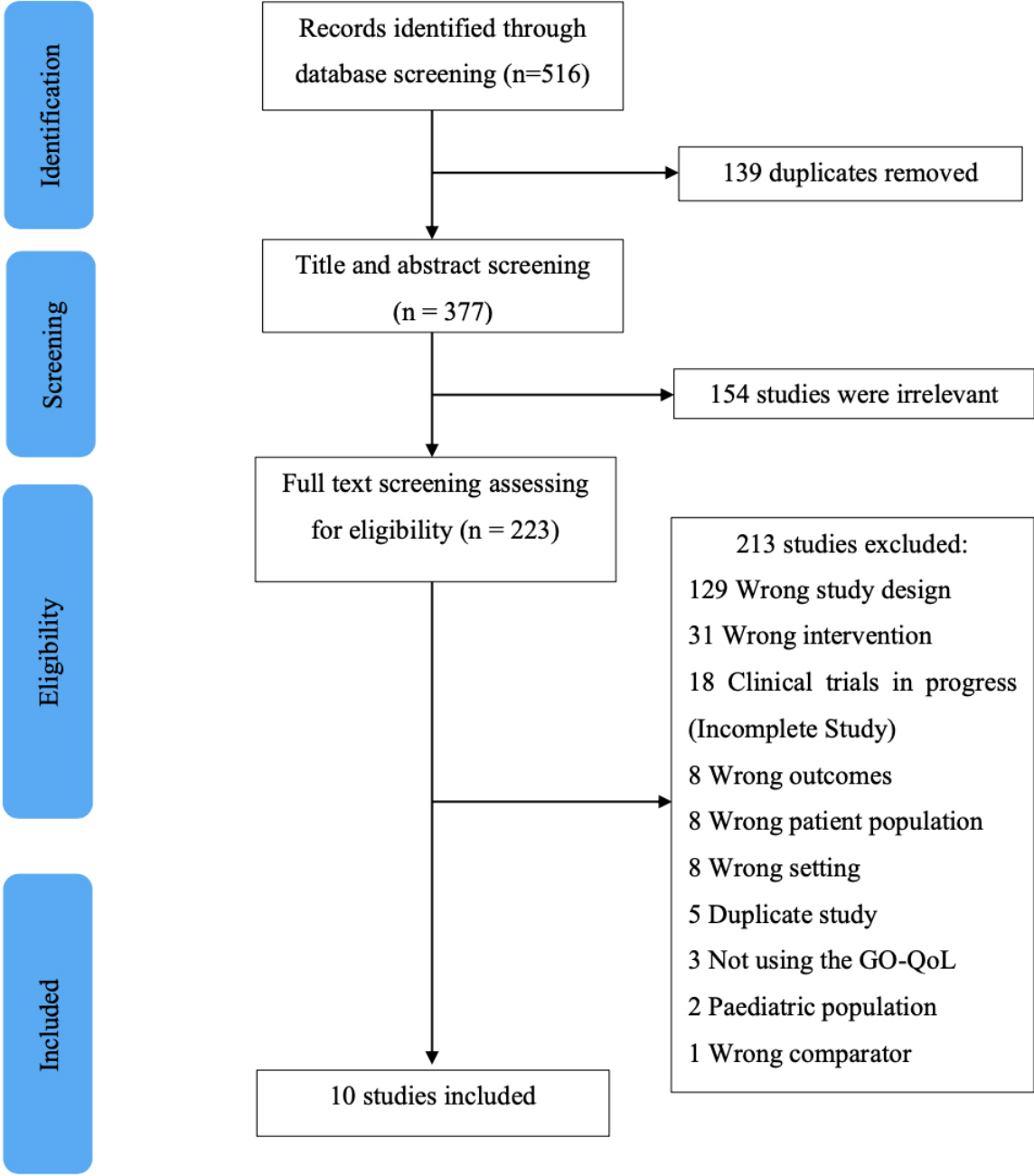

Supplement: Supplementary data [file bmjophth-2023-001515supp001.pdf]
