# Supplementary material for: Graves’ ophthalmopathy: the clinical and psychosocial outcomes of different medical interventions – a systematic review
Source: BMJ Open Ophthalmol. 2024 Jun 17;9(1):e001515. doi: 10.1136/bmjophth-2023-001515 (PMC11184183; doi:10.1136/bmjophth-2023-001515)
Supplement: Supplementary data [file bmjophth-2023-001515supp002.pdf]

Supplementary Tables

Data Search Summary Characteristics of Studies (Table 1)

Quality of Life – Data Extraction Table (Table 2)

Secondary Outcomes Data Extraction Table (Table 3)

Data Search Summary Characteristics of Studies (Table 1)

Study No:	Author & Date	Study Title	Study Design	Number of Participants	Control Group	Treatment type & Administration	Follow up period	Conclusion of Study	Downs & Black Quality Assessment Score /28
1	(Bartalena et al., 2012)	Efficacy and safety of three different cumulative doses of intravenous methylprednisolone for moderate to severe and active Graves' orbitopathy (1)	Multi-centre Randomised double-blind trial	159	No	Cumulative dose of either 2.25g, 4.98g, 7.47g in 12 weekly infusions	6, 12 and 24 weeks	Cumulative dose of 7.47g provides a short-term advantage over lower doses but is associated with slightly greater toxicity	26
2	(Tsirouki et al., 2016)	Clinical and imaging evaluation of the response to intravenous steroids in patients with Graves' orbitopathy and analysis on who requires additional therapy (2)	Prospective cohort study	42	No	Cumulative dose of 4.5g in 12 weekly infusions	12 and 24 weeks	In patients with active GO. IV methylprednisolone is an effective regimen with low adverse effects and recurrence	20
3	(Hoppe et al., 2020)	Predictive Factors for Changes in Quality of Life after Steroid Treatment for Active Moderate-to-Severe Graves' Orbitopathy: A	Prospective follow-up study	100	No	Cumulative dose of 4.5g in 12 weekly infusions	12, 24 and 36 weeks	Treatment with IV steroids is a predictive factor for changes in the GO-QoL questionnaire	18

		Prospective Trial (3)							
4	(Terwee et al., 2001)	Interpretation and validity of changes in scores on the Graves' ophthalmopathy quality of life questionnaire (GO-QOL) after different treatments (4)	Prospective Cohort studies	23	No		24weeks	For invasive therapies, a change of at least 10 points is recommended as a minimal clinically important difference	16
5	(Vannucchi et al., 2021)	Efficacy Profile and Safety of Very Low-Dose Rituximab in Patients with Graves' Orbitopathy (5)	Open-label prospective study	17	No	A single dose of 100mg	4,8,12,16,24, 32, 40, 76 weeks	100mg Rituximab is an effective dose in the management of patients with GO	17
6	(Salvi et al., 2015)	Efficacy of B-cell targeted therapy with rituximab in patients with active moderate to severe Graves' orbitopathy: a randomized controlled study (6)	Double-blind randomised trial	32	No	A cumulative dose of 7.5g IVMP in 12 weekly infusions and RTX 500md or 1000mg X2	12,16,24, 52, 76 weeks	There are better therapeutic outcomes in the management of active GO compared to IV methylprednisolone	23

7	(Perez-Moreiras et al., 2018)	Efficacy of Tocilizumab in Patients with Moderate-to-Severe Corticosteroid-Resistant Graves' Orbitopathy: A Randomized Clinical Trial (7)	Double-blind randomised placebo control trial	32	Yes	8mg/kg intravenous tocilizumab	16, 40 weeks	Tocilizumab offers meaningful improvement in activity and severity in corticosteroid-resistant GO	27
8	(Smith et al., 2017)	Teprotumumab for Thyroid-Associated Ophthalmopathy (8)	Double masked randomised placebo control trial	88	Yes	10mg/kg of teprotumumab for initial infusion the 20mg/kg remaining 7 infusions	6, 12, 18, 24 weeks	Teprotumumab is more effective than placebo in improving proptosis and CAS	26
9	(Douglas et al., 2020)	Teprotumumab for the Treatment of Active Thyroid Eye Disease (9)	Double masked randomised placebo control trial	83	Yes	10mg/kg of teprotumumab for initial infusion the 20mg/kg remaining 7 infusions	6, 12, 18, 24 weeks	Teprotumumab is more effective than placebo in improving proptosis, diplopia, quality of life and CAS	26
10	(Douglas et al., 2022)	Teprotumumab Efficacy, Safety, and Durability in Longer-Duration Thyroid Eye Disease and Re-treatment: OPTIC-X Study (10)	An open-label clinical extension follow-up study	36	No	10mg/kg of teprotumumab for initial infusion the 20mg/kg remaining 7 infusions	4, 12, 24 weeks	Patients with TED of longer disease duration respond similarly to those treated earlier therefore, patients may benefit from additional teprotumumab therapy	18

Quality of Life – Data Extraction Table (Table 2)

N.B: Rows in bold indicate the different time periods when the follow-up appointments were conducted

Study No:	Intervention	Number of Participants	GO-QoL Visual Functioning score at different time periods					GO-QoL Appearance score at different time periods				
			**0 Weeks**	**6 weeks**	**12 Weeks**	**24 weeks**	**P Value**	**0 Weeks**	**6 weeks**	**12 Weeks**	**24 weeks**	**P value**
**1**	2.25g IVMP	53	59 $\pm$ 30	2.1 (-3.4; 7.6)	5.8 (0.3;11.3)		0.11	62 $\pm$ 25	5.2 (0.7; 9.6)	7.6 (3.2;12)		0.003
	4.98g IVMP	54	55 $\pm$ 29	4.7 (-0.7; 10.2)	10.1 (4.7;15.5)		0.001	70 $\pm$ 18	1.9 (-2.5; 6.3)	3.3 (-1.1; 7.6)		0.3
	7.47g IVMP	53	51 $\pm$ 29	7.3 (1.8; 12.9)	12.8 (7.2; 18.3)		<0.0001	62 $\pm$ 23	3.8 (-0.7; 8.2)	9.0 (4.5; 13.5)		<0.0001
**2**	4.5g IVMP	42	4.21 $\pm$ 1.360		5.69 $\pm$ 2.646	8.43 $\pm$ 2.154	<0.001	4.79 $\pm$ 2.926		4.79 $\pm$ 2.926	7.19 $\pm$ 2.998	<0.001
			**0 Weeks**	**12 weeks**	**24 Weeks**	**36 weeks**	**P Value**	**0 Weeks**	**12 weeks**	**24 Weeks**	**36 weeks**	**P value**
**3**	4.5g IVMP	100	66.5 (24.5)	66.1 (23.2)	69.6 (24.9)	72.2 (25.8)	0.04	63.7 (21.6)	71.3 (22.8)	71.1 (25.5)	71.4 (26.3)	0.005
**4**	Orbital Radiotherapy	23	37.0 (20.7)			45.1 (26.9)	0.05	72.0 (18.6)			73.6 (22.7)	0.61

Bello OM, *et al. BMJ Open Ophth* 2024; 9:e001515. doi: 10.1136/bmjophth-2023-001515

**5**	100mg	17	56.4 ± 9.7			52.0 ± 9.8		56.6 ± 6.3			71.6 ± 4.6	
**6**	4.5g IVMP	16							P = 0.39			
	500mg RTX	15		P = 0.058								
			0 Weeks	12 weeks	16 Weeks	40 weeks	P Value	0 Weeks	12 weeks	16 Weeks	40 weeks	P value
**7**	Placebo	17	25.0 (16.7 – 44.0)		35.2 (17.3-58.7)	35.2 (17.3-58.7)		45.0 (30.0-65.0)		17.6 (6.1-41.0)	29.4 (13.2-53.1)	
	Tocilizumab	15	72.2 (38.9 – 83.3)		46.7 (24.8-69.9)	46.7 (24.8-69.9)		50.0 (45.0-55.0)		40.0 (19.9 – 64.2)	33.3 (15.1-58.2)	
			0 Weeks	6 weeks	12 Weeks	24 weeks	P Value	0 Weeks	6 weeks	12 Weeks	24 weeks	P value
**8**	Placebo	45	17.8± 4.3			7.5± 2.7		16.7 ± 3.8			6.6 ± 2.7	
	Teprotumumab	42	16.9 ± 4.4			21.7 ± 2,9	<0.001	17.6 ± 4.5			12.9 ± 2.8	0.10
**9**	Placebo	42		6.33	5.16	1.80			6.33	5.16	1.80	
	Teprotumumab	41		10.18	13.91	17.28	<0.001		10.18	13.91	17.28	<0.001
**10**	Teprotumumab 1st Course	36	70.2	91.7	92.4	95.3		85.0	86.3	89.1	91.7	
	Teprotumumab Retreatment	14	63.8	77.5	79.7	81.3		66.0	68.1	68.1	69.5	

Bello OM, *et al. BMJ Open Ophth* 2024; 9:e001515. doi: 10.1136/bmjophth-2023-001515

Secondary Outcomes Data Extraction Table (Table 3)

Study No:	Intervention	No: of Participants	CAS at different time periods			Proptosis (mm) at different time periods			Diplopia (0/1/2/3) at different time periods			Adverse Events	
			0 Weeks	12 Weeks	P Value	0 Weeks	12 Weeks	P value	0 Weeks	24 Weeks	P Value	Adverse Events	Serious Adverse Events
**1**	2.25g IVMP	53	4	-1.8 ± 1.6	<0.0001	23.3 ± 3.2	-0.8 (-1.2; -0.4)	0.001	17/11/16/9			12 patients	2 patients
	4.98g IVMP	54	4	-2.3 ± 1.4	<0.0001	22.2 ± 3.0	-0.4 (-0.8; -0.01)	0.1	14/11/20/9			18 patients	3 patients
	7.47g IVMP	53	5	-2.7 ± 1.5	<0.0001	22.5 ± 3.8	-0.6 (-1; -0.2)	0.01	15/11/14/12			14 patients	5 patients
			0 Weeks	24 Weeks	P Value	0 Weeks	24 Weeks	P value	0 Weeks	24 Weeks	P Value		
**2**	4.5g IVMP	42	6.05 ± 1.229	1.21 ± 1.440	<0.001								3 patients (1 herpes zoster infection and 2 electrolyte abnormalities)
**3**	4.5g IVMP	100	4			21.8 ± 3.2			32/24/28/16				

Bello OM, *et al. BMJ Open Ophth* 2024; 9:e001515. doi: 10.1136/bmjophth-2023-001515

**4**	Orbital radiotherapy	23				19.4 ± 4.1	18.9 ± 3.7	0.26	4/0/7/10	5/2/6/8	0.32		
**5**	100mg RTX	17	4.6 ± 0.3	1.1 ± 0.2	<0.0001	RE: 23.8 ± 0.7<br>LE: 23.6 ± 1.0	RE: 22.6 ± 0.8<br>LE 23.0 ± 0.9		4/4/2/4			1 case of mild urticaria	1 case of Syndrome of release of cytokines
**6**	4.5g IVMP	16	4.7 ± 0.7	2.3 ± 0.5	<0.006	RE: 22.8 ± 3.3<br>LE: 22.5 ± 3.7		NS	5/4/3/4		NS	7 patients	3 patients
	500mg RTX	15	4.4 ± 0.7	0.6 ± 3	<0.006	RE: 23.2 ± 2.5<br>LE: 23.5 ± 3.5		NS	5/3/5/1		NS	11 patients	2 patients
			0 Weeks	40 Weeks	P Value	0 Weeks	40 Weeks	P value	0 Weeks	40 Weeks	P Value		
**7**	Placebo	17	5 (4.0-6.0)			22 (19.5, 24)	23.2 (19, 24)					17 patients	0 patients
	Tocilizumab	15	5 (5.0-7.0)		>0.05	21 (19.5, 23)	20.7 (18.5, 22)	0.04				13 patients	2 patients

			0 Weeks	24 Weeks	P Value	0 Weeks	24 Weeks	P value	0 Weeks	24 Weeks	P Value		
**8**	Placebo	45	5.2 ± 0.74	-1.85 ± 0.17		23.1 ± 2.9	-0.15 ±0.19		14/19/8/4	18/8/7/6		32 patients	1 patient
	Teprotumumab	42	5.1 ± 0.97	-3.43 ± 0.18	<0.001	-23.4 ± 3.2	-2.46 ± 0.20	<0.001	4/16/7/15	21/4/9/4	<0.001	32 patients	5 patients
**9**	Placebo	42	5.3 ± 0.9	21% of pts achieving CAS 0,1		23.20 ± 3.21	-0.53			Reduction in Diplopia by 1 grade: 29% of pts		29 patients	1 patient
	Teprotumumab	41	5.1 ± 0.9	59% of pts achieving CAS 0,1	<0.001	22.62 ± 3.32	-3.32	<0.001		Reduction in Diplopia by 1 grade: 68% of pts	P=0.001	35 patients	2 patients
**10**	Teprotumumab 1st Course	36	3.6 ± 1.7	65.6% of pts achieving CAS 0,1		23.0 ± 3.1	1-1.5mm improvement					32 patients (86.5%)	3 patients (8.1%)
	Teprotumumab Retreatment	14	3.5 ± 1.6			21.0 ± 4.2	2mm improvement					11 patients (78.6%)	1 patient (7.1%)

N.B: Rows in bold indicate the different time periods when the follow-up appointments were conducted

References

1. Bartalena L, Krassas GE, Wiersinga W, Marcocci C, Salvi M, Daumerie C, et al. Efficacy and safety of three different cumulative doses of intravenous methylprednisolone for moderate to severe and active Graves' orbitopathy. Journal of Clinical Endocrinology and Metabolism. 2012;97(12):4454-63.
2. Tsirouki T, Bargiota A, Tigas S, Vasileiou A, Kapsalaki E, Giotaki Z, et al. Clinical and imaging evaluation of the response to intravenous steroids in patients with graves' orbitopathy and analysis on who requires additional therapy. Clinical Ophthalmology. 2016;10:2277-89.
3. Hoppe E, Lee ACH, Hoppe D, Kahaly GJ. Predictive Factors for Changes in Quality of Life after Steroid Treatment for Active Moderate-to-Severe Graves' Orbitopathy: A Prospective Trial. European Thyroid Journal. 2021;9(6):313-20.
4. Terwee CB, Dekker FW, Mourits MP, Gerding MN, Baldeschi L, Kalmann R, et al. Interpretation and validity of changes in scores on the Graves' ophthalmopathy quality of life questionnaire (GO-QOL) after different treatments. Clinical endocrinology. 2001;54(3):391-8.
5. Vannucchi G, Campi I, Covelli D, Curro N, Lazzaroni E, Palomba A, et al. Efficacy Profile and Safety of Very Low-Dose Rituximab in Patients with Graves' Orbitopathy. Thyroid. 2021;31(5):821-8.
6. Salvi M, Vannucchi G, Curro N, Campi I, Covelli D, Dazzi D, et al. Efficacy of B-cell targeted therapy with rituximab in patients with active moderate to severe graves' orbitopathy: A randomized controlled study. Journal of Clinical Endocrinology and Metabolism. 2015;100(2):422-31.
7. Perez-Moreiras JV, Gomez-Reino JJ, Maneiro JR, Perez-Pampin E, Romo Lopez A, Rodriguez Alvarez FM, et al. Efficacy of Tocilizumab in Patients With Moderate-to-Severe Corticosteroid-Resistant Graves Orbitopathy: A Randomized Clinical Trial. Am J Ophthalmol. 2018;195:181-90.
8. Smith TJ, Kahaly GJ, Ezra DG, Fleming JC, Dailey RA, Tang RA, et al. Teprotumumab for Thyroid-Associated Ophthalmopathy. New England journal of medicine. 2017;376(18):1748-61.
9. Douglas RS, Kahaly GJ, Patel A, Sile S, Thompson EHZ, Perdok R, et al. Teprotumumab for the Treatment of Active Thyroid Eye Disease. N Engl J Med. 2020;382(4):341-52.
10. Douglas RS, Kahaly GJ, Ugradar S, Elflein H, Ponto KA, Fowler BT, et al. Teprotumumab Efficacy, Safety, and Durability in Longer-Duration Thyroid Eye Disease and Re-treatment: OPTIC-X Study. Ophthalmology. 2022;129(4):438-49.
